# Beyond the bench: LGBTQ+ health equity after India’s “no same-sex marriage” verdict

**DOI:** 10.1016/j.lansea.2024.100494

**Published:** 2024-10-08

**Authors:** Sofia Weiss Goitiandia, Akhilesh Agarwal, Smita C. Banerjee, Nirmala Bhoo-Pathy, Chandan Bose, Mahati Chittem, Roop Gursahani, L. Ramakrishnan, Smriti Rana, Naveen Salins, Malar Velli Segarmurthy, Aashiana Thiyam, William E. Rosa

**Affiliations:** a Division of Hospital Medicine, School of Medicine, University of California, San Francisco, CA, USA; b Philip R. Lee Institute for Health Policy Studies, University of California, San Francisco, CA, USA; c Department of Psychiatry, Maimonides Medical Center, Brooklyn, New York, USA; d Department of Psychiatry & Behavioral Sciences, Memorial Sloan Kettering Cancer Center, New York, USA; e Department of Social and Preventive Medicine, Faculty of Medicine, Universiti Malaya, Kuala Lumpur, Malaysia; f Department of Liberal Arts, Indian Institute of Technology Hyderabad, Hyderabad, India; g P.D. Hinduja Hospital, Mumbai, India; h Solidarity and Action Against The HIV Infection in India (SAATHII), Chennai, India; i Strategic Programs and Partnerships, Pallium India Trust, Trivandrum, India; j Department of Palliative Medicine and Supportive Care, Kasturba Medical College Manipal, Manipal Academy of Higher Education, Manipal, India; k Kuala Langat District Health Office, Ministry of Health, Malaysia; l Birmingham and Solihull Mental Health NHS Foundation Trust, Birmingham, UK

**Keywords:** LGBT, LGBTQ+, Lesbian, Gay, Transgender, Same-sex marriage, Legal protection, Health equity, Social justice, Systemic injustice, India

## Abstract

LGBTQ+ people (e.g., lesbian, gay, bisexual, transgender, and queer or questioning people) experience systemic marginalisation and discrimination globally and throughout India. In October 2023, the Indian Supreme Court rejected the legal recognition of same-sex marriage, blocking marriage equality for LGBTQ+ people and contending that the right to marry neither qualifies as a fundamental right accorded by the Indian Constitution nor falls under the Supreme Court’s purview. Although the Supreme Court declared opposition to discrimination based on sexual orientation, its failure to recognise same-sex marriage legally is a substantial obstruction to full LGBTQ+ equality. We propose that the refusal of the Indian legal system to honour same-sex marriage while calling for an end to societal violence and discriminatory behaviour against the LGBTQ+ community is inherently flawed and counterintuitive. Informed by our team’s multidisciplinary orientation as healthcare professionals, researchers, and advocates, we delineate explicit challenges that LGBTQ+ people in India may encounter due to the Supreme Court’s recent ruling. We subsequently put forth a series of interprofessional and intersectoral recommendations to mitigate this decision’s immediate and long-term consequences, providing an actionable path toward LGBTQ+ inclusion, justice, and equity in India.

## Introduction

LGBTQ+ (e.g., lesbian, gay, bisexual, transgender, and queer or questioning people) people worldwide face an ‘epoch of abandonment’ characterised by discriminatory policies; norms that perpetuate shame and secrecy; government-sanctioned violence; stigmatising clinical and research practices; and a lack of inclusion, respect, and safety across health and social care environments.^1^ At the individual level, such inequities frequently translate to disrespectful and insensitive clinical care delivery, mistreatment, traumatisation, and disenfranchisement from health and social care settings.^2^, ^3^, ^4^, ^5^, ^6^, ^7^, ^8^, ^9^, ^10^ At the systemic level, people may face a range of legal penalties for their forced or voluntary disclosure of LGBTQ+ identity.^10^^,^^11^ Globally, 65 jurisdictions criminalise private, consensual, same-sex sexual activity, and 12 countries have jurisdictions that either impose or entail the possibility of the death penalty for private, consensual, same-sex sexual activity.^12^, ^13^, ^14^, ^15^

India, like much of the world, has had a complex and evolving relationship with LGBTQ+ equality across legal, political, social, and health domains (Panel 1).^16^, ^17^, ^18^, ^19^, ^20^, ^21^, ^22^ In a landmark decision in 2018, India’s Supreme Court decriminalised same-sex sexual activity.^23^^,^^24^ The court argued that discrimination based on sexual orientation and the criminalisation of consensual sex between adults in private violated, respectively, individuals’ right to equality and right to privacy, which are protected by the Indian Constitution.^24^, ^25^ Yet, on Oct 17, 2023, the Supreme Court issued a ruling starkly incongruent with its recent decisions. The Court rejected the legal recognition of same-sex marriage, denying petitions from 21 same-sex couples and activists, thereby blocking the path to marriage equality for LGBTQ+ individuals across India.^26^ In their decision, the Court contended that the right to marry does not qualify as a fundamental right accorded by the Indian Constitution and asserted that the authority for establishing marriage equality for same-sex couples falls outside of its purview, resting instead with the parliament. While the Court advocated for an end to discrimination based on sexual orientation, its reluctance to recognise same-sex marriage in the letter of the law has been widely perceived as a setback in the ongoing struggle for full LGBTQ+ equality in India.^27^ There is no legal protection where there is no legal recognition, validity, and inclusion.

Panel 1 **Brief timeline of Indian national events regarding LGBTQ+ equality.**

**Table:** 

Year	Event
1860	Indian Penal Code (Chapter 16, Section 377) declares homosexual intercourse ‘unnatural’ and ‘against the order of nature,’ deeming it a criminal offense.
1947	India gains independence from British empire; homosexuality remains criminalised.
1949	Indian Constitution declares right to equality (Article 14), prohibition of discrimination (Article 15), and right to privacy and personal dignity to all Indian citizens (Article 21); homosexuality remains criminalised.
1990	Homosexuality no longer considered a disease as WHO removes it from the International Classification of Diseases (ICD).
1992	First widely recognised gay rights protest led by activists from the AIDS Bhedbhav Virodhi Andolan (ABVA) outside police headquarters in New Delhi for targeting men from Connaught Place suspected of homosexual activity.
1999	First gay pride parade held in Kolkata named Calcutta Rainbow Pride.
2009	High Court of Delhi decriminalises Section 377 of Indian Penal Code.
2013	India Supreme Court overturns Delhi High Court ruling; turns accountability of amending or repealing Section 377 to Parliament.
2014	Supreme Court dismisses filed review petition to reconsider 2013 decision on Section 377; Supreme Court grants legal recognition to transgender population; Indian Psychiatric Society openly advocates against treating homosexuality as a disease.
2015	Bill introduced to Lok Sabha for decriminalisation of Section 377 and rejected by house vote (71–24).
2016	Supreme Court and five-judge bench investigate Section 377; transgender people first allowed to cast their vote in the “others” category during West Bengal Assembly Elections; homosexuality remains criminalised.
2018	Supreme Court repeals Section 377 of Indian Penal Code and declares the ‘right to health is indispensable’ for LGBTQ+ people.
2019	National Medical Commission (NMC) updates MBBS curriculum to make medical education ‘more comprehensive and relevant to the health needs of society,’ failing to include the sexual health of LGBTQ+ people.
2019	Indian parliament passes the Transgender Persons (Protection of Rights) bill, prohibiting discrimination against transgender people in areas such as education, employment, and housing. It also grants transgender individuals the “right to self-perceived identity,” yet mandates gender-affirming surgery and subsequent registration with the government for official recognition.
2021	Madras High Court recognises ‘queerphobic’ information in undergraduate medical students standardised textbooks; NMC directs that all related unscientific commentary be removed from textbooks (still awaiting execution of such directives).
2023	Supreme Court renders verdict not to legally recognise same-sex marriage.

We propose that the refusal of the Indian legal system to honour same-sex marriage while calling for an end to societal violence and discriminatory behaviour against the LGBTQ+ community is inherently flawed and counterintuitive. This action, coming from a legal entity, not only sends conflicting signals but also undermines efforts toward creating a more inclusive and equitable society–concrete steps the Indian Supreme Court had been consistently demonstrating with previous verdicts.^23^^,^^26^ It is thought that the Court’s failure to enshrine marriage equality is poised to cast a shadow on LGBTQ+ equity in health and social care throughout India, potentially exacerbating the already well-documented challenges in individual and population-level health outcomes experienced by LGBTQ+ individuals nationally.^28^, ^29^, ^30^, ^31^, ^32^, ^33^, ^34^, ^35^

A recent scoping review of LGBTQ+ health in India further emphasises these inequities.^36^ Despite incremental progress, evidence suggests LGBTQ+ health consistently lags behind that of the general population in India. For instance, LGBTQ+ individuals face significant mental health burdens due to societal stigma, discrimination, and violence.^36^ Furthermore, the healthcare system in India is often inaccessible or non-affirming for LGBTQ+ people, exacerbating their health vulnerabilities.^36^ A 2020 study by Pandya and Redcay on transgender health in India, further underscored that transgender individuals are marginalised in multiple ways, from being denied appropriate care to facing systemic barriers in accessing gender-affirming services.^37^ Even in public hospitals, gender-affirmation surgeries are either unavailable or insufficiently provided, forcing transgender people to seek unsafe alternatives.^37^ These structural inequities are compounded by socio-economic exclusion, structurally influenced low health literacy, and a lack of legal protections, further marginalising transgender individuals.^37^

Law-granting institutions cannot claim equity and justice under the shroud of inequitable policies upheld by judgments of the inferiority and unworthiness of LGBTQ+ people. Thus, informed by our team’s multidisciplinary orientation as healthcare professionals, researchers, and advocates, we delineate explicit challenges that LGBTQ+ individuals in India may encounter due to the Supreme Court’s recent ruling. Subsequently, we put forth a series of interprofessional and intersectoral recommendations to mitigate this decision’s immediate and long-term consequences.

## Health and equity ramifications of the Supreme Court ruling

Marriage is a cornerstone institution of society in India and, in many cases, a requirement for social integration and acceptance. The absence of the legal right to marry—or the formal recognition of what same-sex partners consider marriage—delegitimises queer couples, contributing to an environment conducive to the government-sanctioned marginalisation of the Indian LGBTQ+ community; that is, an environment where LGBTQ+ individuals are pushed to the fringes of society through their exclusion from the same opportunities and treatment as others.^38^ In this challenging context, many LGBTQ+ people are likely to find themselves obliged to either conceal their sexual orientation, gender identity, and spousal relationships to maintain respect and acceptance within their social support system or reveal it and risk social exclusion, rejection, and discrimination.^38^ Such choices stand to impose profound and lasting effects on the psychosocial health and well-being of LGBTQ+ individuals, spouses, and family members forced to make them, as outlined by the minority stress hypothesis.^38^^,^^39^

As demonstrated in other countries, social stigmatisation—engendered by the failure to recognise same-sex marriages legally—stands to negatively impact health along myriad axes. In stark contrast, same-sex marriage recognition is associated with positive health and psychological outcomes.^40^, ^41^, ^42^, ^43^, ^44^ Structural bias, systemic discrimination, and harassment are established factors leading to heightened stress and adverse physical and mental health outcomes among LGBTQ+ individuals—a pattern that holds in the Indian context, and which may be exacerbated by the Supreme Court’s recent ruling. Public health threats to LGBTQ+ health may also worsen. For example, the pertinent problem of interpersonal violence may remain particularly challenging to address, with individuals underreporting its occurrence due to shame, stigma, and limited legal recourse given the Court’s decision.^45^ Men and transgender individuals may prove especially vulnerable, given that the Indian criminal code, the *Bharatiya Nyaya Sanhita* (Indian Justice Code), which came into effect in July 2024, also lacks provisions for dealing with the sexual assault of individuals identifying with these gender identities.^46^

Yet, despite these and other challenges, when LGBTQ+ individuals in India face health problems, mental or physical, they are likely to encounter obstacles to accessing timely, high-quality care. Without legal protections such as the acknowledgement of same-sex marriage, many healthcare institutions become psychologically, emotionally, and physically unsafe spaces for those seeking care, particularly if disclosures related to one’s sexual orientation are required. Existing social biases, both explicit and implicit, may be weaponised by healthcare professionals and healthcare delivery frameworks at large, impacting every stage of care and reifying inequities in health. Moreover, these harmful effects extend beyond LGBTQ+ individuals to their families, who will continue to face alienation from loved ones’ healthcare experiences,^7^^,^^8^^,^^47^ as well as the physical and psychological comorbidities associated with disenfranchised grief.^2^^,^^48^

The Supreme Court’s ruling further perpetuates and extends the marginalisation of LGBTQ+ couples from social structures crucial to their health and well-being. Notably, most healthcare insurance policies in India, such as *Ayushman Bharat*, are designed to cover family members, primarily defined along cisheteronormative lines as spouses and children.^49^ Consequently, the Supreme Court’s ruling places same-sex couples, unable to formalise their unions through marriage, at risk of being denied access to essential healthcare insurance as co-dependents.^49^ Similarly, preventing same-sex marriages threatens the potential for other legal protections typically granted to couples upon marriage by the law. These include tax benefits, power of attorney, and rights related to adoption and inheritance, among others. This adds further layers of social and economic stress on LGBTQ+ individuals, which can lead to downstream harm to their health and well-being.

The list of consequences for LGBTQ+ individuals that this ruling will sustain far exceeds those listed herein. Ultimately, legally safeguarding different-sex marriages while denying equivalent recognition to same-sex couples reinforces heteronormativity and a turn toward conservatism in India that has the potential to endanger health and social care outcomes for LGBTQ+ people in the short, medium, and long terms. It underscores to the Indian queer community that they are still perceived as second-class citizens, positioned outside the realm of full equality and justice in the eyes of law.

## Recommendations to foster LGBTQ+-inclusivity in India

To eliminate de facto discrimination within sectors such as healthcare in the absence of adequate legal safeguards, it is incumbent upon professionals spanning various disciplines and types of institutions to take proactive, intentional, strategic, and multi-level measures. Considering this imperative, we present a set of interprofessional and cross-sector recommendations to promote LGBTQ+ -inclusive health and social care in India following the Supreme Court’s ruling.

### Health and social care professionals

Health and social care professionals–from multidisciplinary clinicians to community health workers–must be intentional about cultivating cultural competency and humility in the provision of services for LGBTQ+ persons and families.^50^ Such endeavours include but are not limited to: acquiring an intentional and in-depth understanding of the diverse social and cultural contexts within which LGBTQ+ people in India live; actively seeking education on topics relevant to the LGBTQ+ community; utilising available resources from Indian and global health organisations to promote equitable care delivery; and engaging in community-outreach programs that non-governmental organisations have traditionally led to reach people in their community settings. This expanded outreach will not only benefit India’s LGBTQ+ community but may also aid clinicians in developing a more profound comprehension of the sociocultural dynamics influencing the lives of LGBTQ+ individuals and their interactions with the healthcare system.

Simple and intentional interventions can make significant differences. Through inclusive communication approaches,^51^ clinicians can inquire about patients’ names and pronouns (e.g., he/she/they) and encourage such assessments as standard practice in their institutions. Professionals should also recognise and respect same-sex partners in the clinic, treating them with the same consideration as opposite-sex partners. In cases of serious illness, named same-sex partners ought to be acknowledged as legitimate surrogate decision-makers for the patient.^47^ To further establish a genuinely inclusive healthcare environment, professionals should look to learn and adopt queer-inclusive clinical practices, including in areas such as sexual health assessments and treatments and the provision of mental healthcare services, where LGBTQ+ individuals currently face significant exclusion and stigma from the Indian healthcare system at large.

Indeed, to practice queer-affirmative healthcare, it is crucial to understand the role that healthcare systems have historically played in pathologising the LGBTQ+ community.^52^ Queer-affirmative healthcare should not emerge merely in response to legal recognition but from the necessity to rectify the harms of practices like conversion therapy. Viewing the LGBTQ+ community as an ‘at-risk’ population is reductive and fails to address the deeper systemic issues.^53^ A comprehensive understanding of the underlying causes of vulnerability is essential for truly implementing affirmative and equitable healthcare for LGBTQ+ individuals.

Robust systems-level healthcare training for multidisciplinary professionals and leaders is further essential. This encompasses training to promote sensitive, respectful, and empathic communication strategies and foster inclusive history-taking.^51^^,^^54^ Professionals are also encouraged to call for greater attention to topics relevant to LGBTQ+ populations in medical curricula (see section below), where they are currently only sparsely included.^55^^,^^56^ For example, India’s undergraduate medical curriculum includes the component ‘Attitudes, Ethics, and Communication’ (AETCOM),^57^ within which more comprehensive, nuanced information regarding care provision for LGBTQ+ communities could be incorporated. Developing sections of healthcare curricula such as this could contribute to the early cultivation of understanding and proficiency among future health and social professionals in treating LGBTQ+ people in India.

Studies such as the Harmony intervention underscore the effectiveness of training programs aimed at enhancing healthcare professionals’ knowledge and attitudes, which have subsequently led to improvements in clinical practice.^58^ Through workshops, videos, and group discussions led by community leaders, this particular intervention significantly increased healthcare professionals’ understanding and confidence in providing care to patients from LGBTQ+ populations, which was further corroborated by improved patient satisfaction.^58^

### Health researchers

At the level of health research, investigations to redress the scarcity of data pertinent to the LGBTQ+ community within the Indian context should be prioritised. It is vital to broaden research endeavours into the multiple facets of queer life in India, including understanding the unique health needs of individuals identifying with diverse LGBTQ+ identities. Domains where intensified research would prove advantageous include developing targeted health surveys for LGBTQ+ communities to gather more comprehensive data on the prevalence, incidence, and experiences of different medical conditions,^28^^,^^35^^,^^59^, ^60^, ^61^ as well as undertaking explorations of the lived experiences of queer community members at various tiers of the health system and across different strata of society. These research initiatives should encompass both qualitative and quantitative studies, thoughtfully combined to explore topics relevant to—and ideally driven by—the LGBTQ+ community from multiple, complementary perspectives (i.e., using community-based participatory research approaches). Following the Indian Supreme Court’s ruling, one especially salient focus for such a multi-pronged approach could be the examination of the verdict’s impact on gaps in social services and healthcare access for LGBTQ+ people.

All such research endeavours should be conducted with sensitivity toward India’s historical, cultural, and religious context and address these factors when interpreting gathered data. When feasible, researchers should prioritise collaboration with existing community-based organisations, recognising their wealth of knowledge on LGBTQ+ communities and potential synergies in effecting tangible improvements in care on the ground. To this end, translating research findings into actionable recommendations for policymakers and health and social care professionals also emerges as a critical endeavor. Collaborations with local policy and advocacy organisations represent a crucial avenue for enhancing the visibility and impact of relevant research results.

### Academic institutions

Academic institutions are responsible for ensuring they invest in curriculum development and competence building to specifically address the needs of LGBTQ+ communities across the disciplines. A pivotal step in this direction involves integrating courses on diverse experiences of sexual orientation and gender identity into various programs, with explicit attention also given to LGBTQ+ health in curricula related to medicine and healthcare broadly conceived.^56^^,^^62^ Studies have shown there is a pressing need to develop a comprehensive, pan-India curriculum that can support changing negative attitudes held by some students toward LGBTQ+ populations so that patients receive equitable care. The implementation of curricula sensitising students to the experiences and needs of the LGBTQ+ community could foster increased awareness and acceptance of this population despite the Supreme Court’s ruling on same-sex marriage,^55^^,^^56^ while cultivating a growing cohort committed to advancing LGBTQ+ rights and equity.^56^ The success of such curriculum changes ought to be monitored through continuous evaluation of healthcare students’ attitudes toward LGBTQ+ populations, as has been conducted pre- and post-decriminalisation of same-sex sexual activity following the Supreme Court’s 2018 decision.^63^^,^^64^

Furthermore, academic institutions can actively demonstrate their commitment to LGBTQ+ health and well-being by implementing policies that embrace, rather than ostracise, queer students and employees. These policies should encompass, for example, the establishment of infrastructure facilities that cater to the needs of LGBTQ+ community members, including the provision of gender-neutral restrooms and campus signage. Encouraging the use of inclusive language in official communications, classroom instruction, and written materials would further support a more hospitable environment for LGBTQ+ individuals on Indian campuses.

In conjunction with these policies, academic institutions ought to proactively engage in recruiting and hiring individuals from the queer population, fostering a safe and inclusive environment for their work, training, and professional development. Concurrently, institutions must commit to providing opportunities for a plurality of voices that hold positions of influence. Such a comprehensive approach is essential for cultivating a more inclusive and accepting academic landscape in India, promoting the health and well-being of LGBTQ+ individuals within and beyond its walls.

### Allies and activists

Political mobilisation and building sustained allyship is the life force of health and social equity. Activist groups working on LGBTQ+ issues have tended to work in their own silos and, have rarely been effective at influencing judicial decision-making or appeals. In addition, it is essential to connect civil society organisations, political parties, and faith-based groups to support perspective-taking and develop pragmatic strategies to promote change. LGBTQ+ individuals and communities need to be educated about their constitutional rights so that activists in this space can explore the possibilities of mobilising the voting strengths of the community. Without this, political structures are unlikely to recognise or respect the LGBTQ+ agenda as much as they should.

National-level bodies, like professional associations, other major institutions, and advocacy groups, must be sensitised and mobilised to collaborate and share resources to prevent duplicative efforts and leverage talent. Palliative care and psychosocial welfare organisations should give particular attention to LGBTQ+ patients facing serious and terminal illness, as well as their chosen families, with added emphasis on providing social support and dignified end-of-life and bereavement services.^2^, ^3^, ^4^^,^^47^^,^^51^ Simultaneously, LGBTQ+ individuals must be a focus area for public health education related to advance care planning and its implementation through advance medical directives and healthcare proxy assignments. For those without these documents, recognition of same-sex partners as surrogates and their inclusion in the decision-making hierarchy must be mandated, if need be, through judicial means.

In organisational spheres across sectors, clear Human Resource guidelines must be in place to explicitly outline the unacceptability of harassment toward LGBTQ+ employees, which includes discrimination based on sex, sexual orientation, gender identity, and marital status. Additionally, organisations should actively strive not only to hire LGBTQ+ individuals but also to provide them with competitive benefits. The recent Healthcare Equality Index Resource Guide from the Human Rights Campaign Foundation highlights several critical practices for promoting equity and inclusion for LGBTQ+ employees: (i) recognise LGBTQ+ employees and extend healthcare benefits to domestic partners of all employees, regardless of sexual orientation; (ii) implement an LGBTQ+ -inclusive paid family leave policy that allows paid time off to care for domestic partners, irrespective of biological or adoptive status, along with comprehensive parental leave policies; and (iii) offer bereavement leave benefits that cover the loss of a domestic partner or the immediate family members of the partner.^65^ To date, positive examples of organisational actions include extending benefits to same-sex partners and formally recognising them, as recently demonstrated by private companies like the Godrej Group and Tata Inc. When assessing the inclusivity that is culturally representative of an organisation, there are four planks to bridge health equity divides for LGBTQ+ patients and families (see Panel 2).^66^

Panel 2 **Acquaviva’s four planks of LGBTQ+ inclusive organisations (adapted).66**

**Table:** 

Plank	Considerations
1. Nondiscrimination statement	• Should include key phrases: “gender identity,” “gender expression,” and “sexual orientation.” • Without explicit commitment to nondiscrimination in employment and patient care, LGBTQ+ patients should not feel safe seeking services. • Ensure access to statement on organisation’s website home page; findable via website search box; findable using Google’s search box; printed on every brochure and flyer; available at every phone for people who answer the main phone numbers for the organisation.
2. Employee benefits, orientation, and training	• How organisations treat and train employees informs the tone of the care they provide. • Ensure parity between employees in provision of ALL benefits. • Provide health insurance coverage for gender-affirming procedures and prescriptions for transgender employees. • Include optional questions regarding sexual orientation, sex assigned at birth, and gender identity on employee data collection forms.
3. Intake forms and processes	• People: all employees should be trained in inclusive and respectful care and communication, including phone/switchboard operators, website administrators, social media directors, administrative assistants, triage nurses, admissions/intake staff, volunteers, and all interprofessional clinicians. • Paper: forms and electronic health record fields should include questions, such as: What name would you like to be called? What pronouns do you go by (e.g., he/him, she/her, them/their)? What sex were you assigned at birth? What gender do you identify as now? • Processes: ensure preparation to address emerging events, such as how room assignments are made for a transgender patient admitted inpatient or what happens when someone calls the main number and asks about the organisation’s experience in caring for LGBTQ+ people.
4. Marketing and community engagement	• Visible commitments to equality, inclusion, and nondiscrimination leads to significant reputational benefits. • Website and marketing materials should reflect the diversity of communities being served, make it easy for LGBTQ+ people to see themselves in the images, include images of similarly aged same-gender dyads of male-female dyads used, and avoid visually centreing and privileging the most privileged populations. • Consider advertising in a local LGBTQ+ newspaper, staffing an information booth at an LGBTQ+ pride event, or offering LGBTQ+ specific support or bereavement groups.

Landmark movements have historically sprung from grassroots efforts, and India’s evolving stance on the LGBTQ+ community, while still distant from where it ideally should be, owes much of its progress to the relentless work of grassroots organisations. These tireless efforts have been instrumental in seeking to dismantle the discrimination and marginalisation faced by LGBTQ+ individuals, highlighting the potency of community mobilisation and conveying to the government the demand of much of the population for the protection of the fundamental rights of LGBTQ+ citizens.

In the western region of India, Mumbai's Humsafar Trust has been a steadfast advocate for reproductive health and rights for LGBTQ+ people, forging partnerships with clinics across Mumbai and leaving a lasting positive impact on India’s fight against HIV.^67^ Meanwhile, in the eastern and northeastern regions of India, organisations like Ya_All in Manipur, All Manipur Nupi Maanbi Association (AMANA), and Empowering Trans Ability (ETA) in Manipur, led by youth, have tirelessly mobilised communities in support of LGBTQ+ rights, conducted awareness-raising workshops and online campaigns, and provided crucial crisis support to LGBTQ+ individuals ostracised from their families.^68^ Across the country, from the northern hub of New Delhi's Naz Foundation to the southern metropolis of Bengaluru’s Aravani Art Project, these organisations stand out for their remarkable contributions to the health and well-being of India’s LGBTQ+ communities. Naz Foundation has been instrumental in advocating for LGBTQ+ rights. At the same time, the Aravani Art Project, a collective led by trans and cis women, has been pivotal in reclaiming public spaces through various art initiatives.^69^ Moreover, Aravani nurtures understanding and solidarity between trans and cis women engaged in its art project, exemplifying the transformative impact of community collaboration in fostering societal change. In the domain of healthcare access, Solidarity and Action Against The HIV Infection in India (SAATHII), a pan-India non-profit, was instrumental in advocacy with the Madras High Court in 2021, resulting in orders banning “conversion therapy” (SOGIE change efforts) in the state, leading the National Medical Commission to declare SOGIES change efforts to be professional misconduct, and to amend medical curricula to make them LGBTQ+ affirming.^62^

Queer activism in India, according to Naisargi Dave, is an effect of three affective exercises: “the problematization of social norms, the invention of alternatives to those norms, and the creative practice of these newly invented possibilities.”^70^ From the first known queer protest in India in New Delhi in 1992 to the recent public discourse regarding marriage equality, the nature of queer activism in India has also developed over time to include other marginalised and intersectional identities across gender, caste, class, and region, among others. For example, advocates of LGBTQ+ rights in India, including the Naz Foundation, have begun to pay increased attention to the sexual health and rights of queer migrants who move to cities to escape discrimination and violence in their rural hometowns.

Further, one of the primary accomplishments of LGBTQ+ activism in the past two decades in India overall has been the creation of democratic and community spaces for queer individuals as an alternative to the heteropatriarchal nuclear family. These spaces have evolved from providing shelter against societal stigma to creating a community model for disseminating services like legal aid (VARTA Trust, Kolkata, and QueerTrans–QT Centre, Hyderabad), sexual health guidance (Basera Samajik Sansthan, Noida), and even employment opportunities (Mobbera Foundation, Hyderabad). LGBTQ+ activism in India is part of a broader global effort to showcase how community citizenship addresses the marginalities and vulnerabilities that state citizenship often overlooks.

The significance of the work conducted by all the aforementioned community-based organisations—and myriad others not referred to by name—is more pronounced now than ever, and it is the responsibility of governments and other organisations in positions of power to support their community-engaged work.

### Policymakers

In the aftermath of the Supreme Court’s verdict, policymakers in India have the opportunity—and responsibility—not to perpetuate queerphobic laws but to actively engage in their transformation toward equity. By closely considering research findings, collaborating with community-based organisations, such as those mentioned above, and working alongside advocacy groups, policymakers at various levels—be it local, regional, or national—can foster their understanding of and engagement with the diverse social contexts (e.g., regional origins, religious affiliations, socioeconomic statuses, and more) that shape the lived experiences of LGBTQ+ individuals in India. This knowledge and contact can serve as a foundation for shifts in the social consciousness and the subsequent formulation of more informed, inclusive, and equitable policies.

Furthermore, the Supreme Court’s recent decision posits marriage equality as within the jurisdiction of the legislature. Thus, policymakers at the national level are directly empowered to either extend marriage equality to same-sex couples or evaluate existing legal frameworks, such as domestic partnerships, to afford increased legal recognition and fundamental rights to same-sex couples, including unequivocal access to healthcare insurance, social security benefits, and rights related to adoption and inheritance—much like are afforded to India’s heterosexual married couples. In response to the Supreme Court’s directive to prevent discrimination against LGBTQ+ individuals, policymakers are also urged to enact laws explicitly prohibiting it across various domains of society, with health and social care crucial among them.

Beyond enacting favourable legislation, policymakers in India must establish effective enforcement systems to address infringements of the law, thereby ensuring that efforts toward LGBTQ+ equality are not only principled but pragmatic. This is particularly critical considering how often queer individuals in India face threats to their health—and even lives—due to their sexual orientation and gender identity or expression, as has been outlined earlier.

Though we are under no pretences about the existing hostility toward LGBTQ+ communities within certain factions of the current Indian government, we recognise that their perspective is not universal. A growing number of policymakers in India actively support the LGBTQ+ cause. We urge them and their allies to continue providing a platform for queer voices to be heard and amplified within political structures, including the legislature, while supporting affirmative steps toward instigating necessary change. Post-Supreme Court ruling, the time for action is now.

## Conclusion

India cannot move forward in queer rights until there is comprehensive public acceptance of LGBTQ+ individuals. To foster acceptance, there needs to be visibility of LGBTQ+ individuals; however, in the absence of a safe and welcoming environment, “coming out” and related increased visibility necessarily become secondary; ensuring one’s survival takes precedence. Clinicians, researchers, academic institutions, allies, activists, and policymakers are all key stakeholders in not only promoting health and social equity for LGBTQ+ patients, partners or spouses, families, and communities but also in identifying and dismantling discriminatory policies and care services. The Supreme Court’s decision echoes the implicit and misguided social premise that same-sex marriages are undeserving of respect or validation, as are the human beings involved in them. To rectify the implications of their decision and procure safe environments for people-centred care, all stakeholders must prioritise LGBTQ+ inclusion, question assumptions, and challenge extant health systems and structures. Although same-sex marriage may remain illegal and unrecognised in India, we can all take active parts to ensure the health equity fallout of this decision is tackled head-on.

## Contributors

Conceptualisation: WER. Writing—original draft: All authors. Writing—review and editing: All authors.

## Declaration of interests

SCB and WER are both partially funded by 10.13039/100000054NCI/10.13039/100000002NIH Comprehensive Cancer Centre award number P30 CA008748. The authors have no other conflicts of interest to disclose.
